# Effect of dietary supplementation of β-mannanase on growth performance, carcass characteristics, excreta microflora, blood constituents, and nutrient ileal digestibility in broiler chickens

**DOI:** 10.5713/ab.20.0355

**Published:** 2021-01-01

**Authors:** Mohsen Mohammadigheisar, Victoria L. Shouldice, Balamuralikrishnan Balasubramanian, In Ho Kim

**Affiliations:** 1 Department of Animal Resource and Science, Dankook University, Cheonan 31116, Korea; 2 Department of Animal Biosciences, University of Guelph, Guelph, ON, N1G2W1, Canada; 3 Department of Food Science and Biotechnology, College of Life Science, Sejong University, Seoul 05006, Korea

**Keywords:** Broiler, Carcass Characteristic, Dehulled Soybean, Ileal Digestibility

## Abstract

**Objective:**

The aim of the present study was to investigate the effects of dietary supplementation of β-mannanase on growth performance, carcass characteristics, excreta microflora, blood constituents, and nutrient digestibility in broiler chickens.

**Methods:**

A total of 680 one-d-old Ross 308 (as hatched) broiler chickens were used in a 35-d growth assay. Chicks were sorted into pens with 17 birds/pen and 10 pens/treatment. Treatment diets were contained either 44% or 48% crude protein (CP) soybean meal (SBM) with or without β-mannanase.

**Results:**

Using SBM containing 48% CP led to an improvement (p<0.05) in feed conversion ratio (FCR) from d 1 to 14. Addition of β-mannanase to the diets significantly improved body weight gain (BWG) and FCR from d 1 to 14. During overall experimental period, BWG was affected (p<0.05) by CP level of SBM and inclusion of β-mannanase, but FCR and feed intake were not affected. Carcass characteristics were not influenced by treatment diets. The results showed that digestibility of dry matter (DM), nitrogen (N), and energy was not affected by CP level of SBM and/or inclusion of β-mannanase. Among essential amino acids (EAA) apparent digestibility of valine, methionine, and leucine improved (p<0.05) by the addition of β-mannanase to the diets. The results demonstrated that ileal digestibility of DM, N, and energy was not affected by treatment diets. Among EAA, the ileal digestibility of valine and arginine was higher (p<0.05) in the diets containing 48% CP SBM and/or β-mannanase. Excreta *Lactobacillus* count increased (p<0.05) by the addition of β-mannanase to the diets. Blood urea nitrogen, creatinine, and total protein level were not affected by treatments.

**Conclusion:**

Feeding chickens with diets containing 44% CP SBM resulted in detrimental effects on growth performance and digestibility of nutrients, but addition of β-mannanase to the 44% CP diet improved the growth performance of chickens without any effects on carcass characteristics.

## INTRODUCTION

Soybean meal (SBM) is one of the most used protein sources in poultry and swine nutrition around the globe. Relatively high levels of crude protein (CP), an excellent profile of amino acids (AA), and high AA digestibility are some reasons for the universal acceptability of SBM [1]. Similar to most plant-derived feedstuff, SBM has some anti-nutritional factors such as trypsin inhibitor (TI) and non-starch polysaccharides (NSP) that can limit the bioavailability of SBM protein. Heat processing is one effective approach to eliminate or reduce the level of TI [1]. It has been reported that higher content of fiber in processed SBM leads to lower growth performance of broiler chickens [2]. Researchers stated that there is a relationship between water-soluble xylose content of SBMs and improvement in weight gain when sunflower meal is used to replace some SBM [3]. It has been reported that SBM contains 1.3% β-mannans [4]. β-Mannan, classified as a soluble NSP, is composed of sequential units of mannose complexed with galactose or glucose linked to β-mannan backbone [4–7]. Due to the lack of endogenous NSP degrading enzymes in poultry and swine, NSP cannot be hydrolyzed and digested in their gut [8]. The only known way to digest them is through bacterial fermentation. It has been well documented that the addition of exogenous enzymes such as β-mannanase, xylanase, α-amylase, and β-glucanase can alleviate the anti-nutritional effects of NSP [9,10]. Previously, other researchers reported the anti-nutritional effect of soybean oligosaccharides and soluble NSP in weaning piglets [11,12]. Reducing the fiber content of feedstuff and consequently reducing the concentration of NSP can also be beneficial in improving the performance of poultry and swine. It has been suggested that removing soluble NSP and oligosaccharide by ethanol/water extraction improved intestinal health and growth performance in weaning piglets [13]. Dehulling is another possible approach to reduce the NSP content of SBM. Researches have shown that NSP can increase the viscosity of digesta, modify the physiology of gastrointestinal tract, and change the ecosystem in the gut [14]. It has been stated that a commonly found NSP in feedstuff is β-mannan that can negatively affect the performance of animals [6]. However, there is some evidence showing that β-mannan can stimulate the innate immune system and is potentially capable of stimulating nonproductive energy draining innate immune responses [15–17]. Some researchers have suggested that dehulled SBM contains a higher concentration of protein and a lower level of fiber compared to hulled SBM [18]. Therefore, the objective of this study was to investigate the effect of using dehulled SBM and supplementing the diets with β-mannanase on growth performance, carcass characteristics, excreta microflora, blood constituents, and nutrient ileal digestibility in broiler chickens.

## MATERIALS AND METHODS

### Animal care

The experimental procedures used in this trial were approved by the Animal Care and Use Committee of Dankook University (approval number DK-1-1516).

### Animals, diets, and management

In this experiment, 680 one-d-old Ross 308 (as hatched) broiler chicks with an average initial body weight (BW) of 43±0.54 g were used in a 35-d experimental period. The chicks were sorted into pens (17 birds per pen and 10 pens per treatment) and housed in battery cages (1.55×0.75×0.55 m/cage), in an environmentally controlled room (32°C to 24°C and 65% relative humidity). They were allowed free access to feed and water during the experiment. Each cage was equipped with two feeders (one feeder in each side) and two nipple drinkers.

The treatment diets were: i) T1, 44% CP SBM; ii) T2, T1 +0.05% β-mannanase; iii) T3, 48% CP SBM, and iv) T4, T3+0.05% β-mannanase. Four isocaloric diets were formulated to meet Ross 308 nutrient recommendations (Table 1) [19]. Enzyme preparation used in this study was provided by a local manufacturer (CTC Bio Inc., Seoul, Korea). It was produced by using *Bacillus subtilis* (WL-1) grown on Luria broth at guaranteed activity of 800 international unit/g (reducing sugar assay). One IU is defined as the quantity of enzyme required to generate 1 μmol of reducing sugar/min, at pH 6.0 and 50°C [19]. According to the manufacturer’s information, β-mannanase was purified from crude solution produced by *Bacilli* optimized to produce only β-mannanase.

The BW and remained feed were measured on d 0, 10, and 35 to calculate body weight gain (BWG), feed intake (FI), and feed conversion ratio (FCR). Mortality was recorded daily to allow the further correction of FCR. The finisher diet contained 0.2% of chromium oxide (Cr_2_O_3_) as marker to allow determination of apparent retention (AR) and digestibility of components.

### Sampling

Excreta samples for AR assay; 10 samples per treatment) were collected in 3 consecutive days from d 32 to 35 and stored at −20°C until further analysis. On d 35, 160 birds (40 birds per treatment) were randomly selected (10 birds per treatment were bleed before euthanasia, samples were collected from a wing vein into vacuum tubes) and euthanized by cervical dislocation. Breast muscle, liver, abdominal fat, and empty gizzard were weighed. Ileal digesta (portion of the small intestine from Meckel’s diverticulum to approximately 1cm proximal to the ileo-cecal junction) were collected into sample bags and placed on ice and stored at −20°C.

### Sample processing and analyses

Hulled and dehulled SBM were analyzed for CP (method 990.03) [21], ether extract (method 2003.06) [21], ash (method 942.05) [21], acid detergent fiber (ADF; method 973.18) [21], and neutral detergent fiber (NDF) [21]. To determine mannan content, test ingredients were hydrolyzed with 72% (w/w) H_2_SO_4_ for 1 h. Samples were then diluted with distilled water to final concentration of H_2_SO_4_ at 1N and incubated at 121°C for 45 min. Mannan contents in the hydrolysates were determined using an evaporative light scattering detector and a Shodex sugar column SP0810 (8.0 mm×300 mm; Showa Denko K.K., Tokyo, Japan; Table 2) [23].

Ileal digesta samples were weighed and then freeze-dried at −53°C for 72 h by freeze-dryer (FD5510, Freeze Dryer, Ilshin Lab, Dongducheon, Korea) after which they were finely ground to the size that could pass through a 1 mm screen. Chromium was analyzed by UV absorption spectrophotometry (Shimadzu, UV-1201, Shimadzu, Kyoto, Japan) [24]. The excreta samples were pooled and homogenized, and the moisture content determined by placing in an oven at 80°C for 48 h. Diet samples and air-dried excreta samples were finely ground. All the samples were analyzed for dry matter (DM, method 930.15) [21], N (Leco N analyzer; FP-528; Leco, Saint Joseph, MI, USA), gross energy (GE; IKA bomb calorimeter, C5000; IKA Works, Wilmington, NC, USA), and Cr_2_O_3_. The AR of components was calculated [25] as followed:

AR %=[([NT/Cr] diet×[NT/Cr] excreta)/(NT/Cr) diet]×100

Where (NT/Cr) diet is ratio of component and chromium oxide in the diet, and (NT/Cr) excreta is ratio of component and chromium oxide in excreta. Component can be DM, N, or GE. The relative weights of breast meat, abdominal fat, and organs were expressed as percentage of live BW.

Serum samples were obtained by spinning blood samples at 3,000×*g* for 15 min at 4°C. Blood urea nitrogen (BUN), creatinine, and total protein concentrations were measured using an automatic biochemistry blood analyzer (Hitachi 747, Hitachi, Tokyo, Japan).

### Statistical analysis

All data were subjected to statistical analysis in a completely randomized design, with a 2×2 factorial arrangement using general linear model procedures of SAS [26]. Each pen was used as an experimental unit. The main effects included different source of SBM and enzyme inclusion. The mean values and standard errors were reported. Probability values of less than 0.05 were considered as statistically significant.

## RESULTS

### Growth performance

Growth performance results are summarized in Table 3. Using different SBM containing 44% CP or 48% CP in the diets did not significantly affect BWG from d 1 to 10. But supplementing the diets with β-mannanase improved (p< 0.05) BWG during the starter phase. The results showed that feeding chickens with the diet containing SBM 48% CP significantly improved (p<0.05) FCR. It was also observed that the addition of β-mannanase to the diets improved (p = 0.05) FCR from d 1 to 10. The data showed that feeding the chickens with the diets containing SBM with 44% CP resulted in lower (p = 0.02) BWG but addition of β-mannanase to the diets improved (p = 0.02) BWG during the overall experimental period. Both FI and FCR were not significantly affected by treatment diets.

### Relative organ weights

Results of the relative organ weights and breast meat yield are summarized in Table 4. The results showed that using different SBM containing 44% or 48% CP or adding β-mannanase to diets did not affect the relative weights of abdominal fat, liver, gizzard, and breast meat yield.

### Nutrient digestibility

Results of AR of DM, N, and gross energy are shown in Table 5. The AR of DM, N, and GE were not significantly affected by treatment diets. Apparent ileal digestibility analysis also showed that DM, N, GE, and total essential amino acids (EAA) were not significantly influenced by dietary treatments. Among EAA valine and arginine showed a tendency (p<0.10) to be affected by treatments. Feeding the chickens with the diets composed of dehulled SBM and adding β-mannanase to the diets improved (p<0.05) the apparent ileal digestibility of valine and arginine (Table 5).

### Excreta microbiota and blood constituents

The results of excreta microbiota assay presented in Table 4 showed that regardless of the CP level of SBM in the diet, addition of β-mannanase significantly increased (p = 0.002) the count of excreta *Lactobacillus*; these results also indicated that there was a trend (p = 0.06) in reducing the count of excreta *Escherichia coli* (*E. coli*).

Blood sample analysis showed that using different sources of SBM did not significantly affect the concentration of blood urea nitrogen, creatinine, and total protein. Supplementing diets with β-mannanase tended to increase (p = 0.06) the level of serum total protein. The levels of BUN and creatinine were not significantly affected by the addition of β-mannanase to the diets.

## DISCUSSION

In general enzymes are added to feeds in order to augment low levels of natural endogenous enzymes or to add novel enzymatic systems not naturally produced by the bird [27]. Several researchers have reported that supplementing enzymes in the diets of broiler chickens improved overall performance [28–30]. It was also reported that replacing hulled SBM (44% CP) with de-hulled SBM (48% CP) led to an improvement in the performance of pigs [18]. Our findings in the current study showed that feeding broiler chickens with diets containing hulled or dehulled SBM supplemented with exogenous enzyme improved BWG at the starter phase. The results of the current study agreed with the results of previous studies showing beneficial effects of adding NSP degrading enzymes to the diet of broiler chickens [31]. Previous researchers reported that broiler chickens fed the diet supplemented with β-mannanase showed 3.5% higher BW compared to the birds fed diets without β-mannanase [32]. It has been reported that the content of β-mannan in broiler chickens starter diet containing 35% dehulled SBM is approximately 0.44% [6]. The results of previous studies suggested that simple sugars released from this amount (0.44% β-mannan) would not be enough to improve the performance of chickens [32]. They concluded that β-mannanase supplementation improved the apparent metabolizable energy (AME) of diets. The results of the current study were consistent with the findings of previous researchers showing that adding β-mannanase to the diet of broiler chickens improved BWG during the starter phase [4,6,33].

It has been reported that highly viscous nature of β-mannans is the main reason for their adverse effect on the function of digestive system. The high viscous nature of β-mannans results in lowering gastric emptying rate, restricting the contact of nutrients with absorptive epithelium [34].

Our findings showed that the addition of β-mannanase to the diets composed of different sources of SBM did not affect the retention of DM, N, and GE. The content of β-mannans in hulled and dehulled SBM was 1.7% and 1.6%, respectively, indicating that there was not a considerable difference between the content of substrate for β-mannanase. The low amount of β-mannans as the substrate of β-mannanase might be the reason why the retention of nutrients was not significantly improved. Previous researchers reported that mannans content of guar gum in broiler feed significantly increased intestinal supernatant viscosity [6]. It has been suggested that the effect of β-mannanase in reducing intestinal viscosity was the solution to overcome the anti-nutritional effect of mannans [35]. Supplementing the diets with β-mannanase resulted in preparing a suitable environment for the growth of *Lactobacillus*. It has been observed that supplementing broiler chicken diets with β-mannanase improved AR and apparent ileal digestibility of DM during the starter phase [32]. It can be speculated that the addition of β-mannanase to the diet of broiler chickens led to the generation of mannan-oligosaccharides, mannotriose, and mannobiose, as well as a small amount of mannose [32] which might have supported the improvement in BWG during starter phase.

Other researchers supplemented the diet of broiler chickens with β-mannanase and reported that addition of β-mannanase did not influence the blood proteins (albumin α1, albumin α2, albumin β, and γ-globulins) [36]. The results of current study agreed with previously published data. We did not observe any significant effect on BUN showing that source of SBM and/or addition of β-mannanase to the diets did not influence the efficiency of nitrogen which is consistent with the results of previous studies [31,37]. In a trial with growing pigs fed with diets containing hulled or dehulled SBM with or without β-mannanase the results showed that the count of excreta microbiota was not influenced by the source of SBM. They reported that addition of β-mannanase reduced the count of excreta *E. coli* [38].

## CONCLUSION

In conclusion, the results of the current study showed that supplementing the diet of broiler chickens with β-mannanase improved their growth performance. Our findings indicated that dietary β-mannanase could improve the gut environment in favor of beneficial bacteria such as *Lactobacillus* while reducing the activity of *E. coli* which ultimately resulted in improving feed efficiency and BWG especially during the starter phase which is the critical stage of commercial broiler chickens.

## Figures and Tables

**Table 1:** Ingredient composition of experimental diets (as-fed basis)

Items	Starter (d 1 to 10)		Finisher (d 11 to 35)	
	
	Hulled SBM 44%	Dehulled SBM 48%	Hulled SBM 44%	Dehulled SBM 48%
Ingredient (%)				
Corn	42.87	46.43	47.94	49.12
Soybean meal, 44%	37.15	-	26.33	-
Soybean meal, 48%	-	33.97	-	28.28
Pork meal (60%)	5.00	5.00	5.00	5.00
Wheat	8.66	8.66	10.00	10.00
Corn gluten meal (60%)	-	-	4.31	0.65
Soybean oil	3.48	2.95	3.99	4.47
Monocalcium phosphate	0.48	0.51	0.31	0.29
Limestone	0.68	0.69	0.51	0.50
NaCl	0.27	0.24	0.13	0.14
NaHCO_3_	0.29	0.33	0.48	0.47
L-lysine HCL (78%)	0.22	0.31	0.26	0.24
DL-methionine (99%)	0.35	0.37	0.25	0.31
L-threonine (98%)	0.15	0.15	0.10	0.13
Vitamin premix^1)^	0.20	0.20	0.20	0.20
Mineralpremix^2)^	0.20	0.20	0.20	0.20
Chemical composition				
AME (MJ/kg)	12.56	12.56	13.19	13.19
CP (%)	24.00	24.00	22.00	22.00
dLys (%)	1.28	1.28	1.11	1.11
dMet+Cys (%)	0.95	0.95	0.85	0.85
Na (%)	0.21	0.21	0.21	0.21
Ca (%)	0.96	0.96	0.84	0.84
aP (%)	0.48	0.48	0.42	0.42

AME, apparent metabolizable energy; CP, crude protein; aP, available phosphorus.

1) Provided per kg of complete diet: 11,025 IU vitamin A; 1,103 IU vitamin D_3_; 44 IU vitamin E; 4.4 mg vitamin K; 8.3 mg riboflavin; 50 mg niacin; 4 mg thiamine; 29 mg d-pantothenic; 166 mg choline; 33 μg vitamin B_12_.

2) Provided per kg of complete diet: 12 mg Cu (as CuSO_4_·5H_2_O); 85 mg Zn (as ZnSO_4_); 8 mg Mn (as MnO_2_); 0.28 mg I (as KI); 0.15 mgSe (as Na_2_SeO_3_·5H_2_O).

**Table 2:** Analyzed composition of hulled and dehulled soybean meals

Items (%)	Hulled SBM^1)^	Dehulled SBM^1)^
Crude protein	44.6	47.7
Ash	6.5	6.0
Crude fat	1.9	1.5
ADF	7.51	5.15
NDF	13.93	8.09
NFE	31.7	32.0
Beta mannan	1.7	1.6

ADF, acid detergent fiber; NDF, neutral detergent fiber; NFE, nitrogen free extract.

1) Hulled SBM, soybean meal (44% crude protein); Dehulled SBM, soybean meal (48% crude protein).

**Table 3:** Effect of dietary supplementation of β-mannanase on growth performance in broilers

Items	H-SBM^1)^		DH-SBM^1)^		SEM	p-value		
		
	− ENZ	+ ENZ	− ENZ	+ ENZ		SBM	ENZ	SBM×ENZ
d 1 to 10								
BWG (kg/bird)	0.284	0.289	0.287	0.293	2.41	0.19	0.04	0.96
FI (kg/bird)	0.297	0.297	0.294	0.295	1.35	0.09	0.95	0.66
FCR	1.046	1.026	1.025	1.008	0.01	0.04	0.05	0.91
d 11 to 35								
BWG (kg/bird)	1.677	1.685	1.691	1.707	13.34	0.18	0.36	0.76
FI (kg/bird)	2.704	2.696	2.706	2.705	24.46	0.83	0.86	0.89
FCR	1.615	1.601	1.601	1.586	0.02	0.47	0.47	0.98
Overall								
BWG (kg/bird)	1.961	1.974	1.978	2.000	12.34	0.09	0.17	0.74
FI (kg/bird)	3.001	2.993	3.000	2.999	24.49	0.94	0.86	0.87
FCR	1.530	1.518	1.517	1.500	0.02	0.29	0.35	0.93

SEM, standard error of mean; BWG, body weight gain; FI, feed intake; FCR, feed conversion ratio.

1) H-SBM, hulled soybean meal (44% crude protein); DH-SBM, dehulled soybean meal (48% crude protein); ENZ, enzyme (inclusion level: 0.05%).

**Table 4:** Effect of dietary supplementation of β-mannanase on organ weights, blood profile, and excreta microbial counts in broiler chickens

Items	H-SBM^1)^		DH-SBM^1)^		SEM	p-value		
		
	−ENZ	+ ENZ	−ENZ	+ ENZ		SBM	ENZ	SBM×ENZ
Relative weight of organs (%)								
Breast muscle	17.48	17.63	17.60	17.60	0.59	0.95	0.90	0.90
Liver	3.03	3.02	2.98	3.03	0.13	0.86	0.89	0.83
Abdominal fat	1.46	1.55	1.53	1.49	0.10	0.98	0.78	0.49
Gizzard	1.26	1.24	1.25	1.27	0.05	0.82	0.99	0.71
Blood profile								
BUN (mmol/L)	2.88	2.91	3.01	2.93	0.19	0.68	0.91	0.76
Creatinine (mmol/L)	0.19	0.19	0.17	0.17	0.02	0.28	0.78	0.88
Total protein (g/L)	4.3	4.5	4.2	4.7	0.20	0.87	0.06	0.46
Excreta microbial counts (log_10_ cfu/g)								
*Lactobacillus*	7.61	7.67	7.63	7.73	0.02	0.12	0.002	0.29
*Escherichia coli*	6.55	6.46	6.52	6.44	0.04	0.47	0.06	0.93

SEM, standard error of mean; BUN, blood urea nitrogen.

1) H-SBM, hulled soybean meal (44% crude protein); DH-SBM, dehulled soybean meal (48% crude protein); ENZ, enzyme (inclusion level: 0.05%).

**Table 5:** Effect of dietary supplementation of β-mannanase on apparent retention of components and apparent ileal digestibility of essential amino acids in broiler chickens

Items	H-SBM^1)^		DH-SBM^1)^		SEM	p-value		
		
	− ENZ	+ ENZ	− ENZ	+ ENZ		SBM	ENZ	SBM×ENZ
Components (%)								
Dry matter	75.4	74.8	74.8	75.2	0.40	0.70	0.83	0.26
Nitrogen	66.9	67.3	67.4	67.6	1.32	0.79	0.84	0.93
Energy	70.6	71.1	71.1	71.6	0.46	0.28	0.31	0.99
Essential amino acids (%)								
Arg	85.2	85.7	85.6	86.1	0.24	0.11	0.07	0.99
His	84.2	84.3	84.3	84.5	0.49	0.85	0.78	0.95
Ile	81.3	82	82.1	82.4	0.35	0.13	0.15	0.50
Leu	82	82.8	82.9	83.2	0.51	0.2	0.31	0.68
Lys	82.4	82.6	82.7	82.8	0.53	0.67	0.71	0.96
Met	88	89.1	89.1	89.2	0.63	0.34	0.33	0.49
Phe	83.1	83.3	83.4	83.5	0.51	0.69	0.77	0.90
Thr	74.1	74.4	74.4	74.7	0.91	0.79	0.75	0.99
Trp	78.2	78.6	78.5	78.9	0.88	0.75	0.69	0.97
Val	79.6	80.4	80.4	80.8	0.21	0.01	0.01	0.39
TEAA	82.0	82.5	82.5	82.8	0.35	0.25	0.27	0.73

SEM, standard error of mean; TEAA, total essential amino acids.

1) H-SBM, hulled soybean meal (44% crude protein); DH-SBM, dehulled soybean meal (48% crude protein); ENZ, enzyme (inclusion level: 0.05%).
